# From Gut to Gray Matter: A Case Report of Posterior Reversible Encephalopathy Syndrome in a Pediatric Patient With Celiac Disease

**DOI:** 10.1002/ccr3.70260

**Published:** 2025-03-20

**Authors:** Hashim Salar, Khizer Masroor Anns, Musa Salar, Faheemullah Khan, Muhammad Aman, Uffan Zafar, Izaz Ahmad, Sundas Basharat, Rehana Murad, Khizar Salar, Shayan Sirat Maheen Anwar

**Affiliations:** ^1^ Medical College The Aga Khan University Karachi Pakistan; ^2^ Department of Radiology Cleveland Clinic Cleveland Ohio USA; ^3^ Department of Radiology The Aga Khan University Hospital Karachi Pakistan; ^4^ Pak International Medical College Peshawar Pakistan; ^5^ Islamabad Diagnostic Center (IDC) Islamabad Pakistan; ^6^ Department of General Surgery University of New Mexico Albuquerque New Mexico USA

**Keywords:** case report, celiac disease, fatal outcome, pediatric, posterior reversible encephalopathy syndrome, PRES

## Abstract

Posterior reversible encephalopathy syndrome (PRES) is a rare neurological condition characterized by vasogenic edema, primarily affecting the posterior cerebral hemispheres. Although typically reversible with prompt treatment, PRES can lead to severe complications if not recognized early. This report presents an unusual and fatal case of PRES in a pediatric patient with celiac disease, a combination that is rarely documented in medical literature. A 9‐year‐old child with a history of celiac disease and dermatitis herpetiformis presented to the Emergency Room with a two‐month history of vomiting and loose stools, accompanied by a recent 20‐min seizure. Initial examination revealed pallor, emaciation, drowsiness, and a generalized rash. The patient was tachycardic, hypertensive (blood pressure 150/100 mmHg), and had an oxygen saturation of 65%. Neurological examination showed a glasgow coma scale (GCS) score of 10/15, increased muscle tone, and heightened reflexes. CT and MRI scans revealed intraparenchymal and subarachnoid hemorrhages. Despite aggressive management including intubation, antihypertensive therapy, anticonvulsants, and broad‐spectrum antibiotics, the patient's condition deteriorated rapidly. Complications included pneumoperitoneum and eventual cardiac arrest, leading to the patient's death. This case highlights the potential for severe, life‐threatening complications of PRES in pediatric patients with underlying autoimmune conditions such as celiac disease. It underscores the importance of considering PRES in the differential diagnosis for children with celiac disease presenting with neurological symptoms, even in the absence of typical radiological findings. The case also illustrates the need for further research into the relationship between celiac disease and PRES to improve outcomes in similar cases.

**Summary**

- Celiac disease can be a significant risk factor for posterior reversible encephalopathy syndrome (PRES) in pediatric patients.
- Clinicians should maintain a high index of suspicion for PRES when encountering children with celiac disease who present with persistent hypertension and neurological symptoms.
- Prompt recognition and management are crucial to prevent severe complications and improve outcomes.

## 1 Introduction

Posterior reversible encephalopathy syndrome (PRES), also known as reversible posterior leukoencephalopathy syndrome (RPLS), is a clinico‐radiological condition characterized by a constellation of neurological symptoms including visual disturbances, seizures, headaches, and altered mental status [1]. First described by Hinchey et al. in 1996 [2], it is a neurotoxic state that arises due to the failure of the posterior circulation to autoregulate in response to acute changes in blood pressure, leading to hyperperfusion and resultant vasogenic edema, typically without infarction [3]. Although the exact pathophysiology remains debated, two leading theories propose either a failure of cerebral autoregulation leading to hyperperfusion or endothelial dysfunction caused by circulating toxins [4].

PRES has been associated with various conditions, including hypertensive emergencies, (pre)eclampsia, autoimmune disorders, and exposure to cytotoxic or immunosuppressive medications [1]. The syndrome affects individuals across all age groups, with a preponderance in young to middle‐aged adults and a female predominance [5]. Neuroimaging, especially magnetic resonance imaging (MRI), plays a crucial role in diagnosis, typically revealing a parieto‐occipital pattern of vasogenic edema [6]. However, atypical distributions involving the frontal lobes, cerebellum, or brainstem have been reported [7]. While generally considered reversible, PRES can lead to severe complications, including status epilepticus, cerebral ischemia, and intracranial hemorrhage, potentially resulting in long‐term neurological sequelae or, in rare cases, mortality [8].

This case report presents a complex presentation of PRES in a 9‐year‐old child with a history of celiac disease and dermatitis herpetiformis. The case highlights the potential severity of PRES in pediatric patients with underlying autoimmune conditions and underscores the importance of prompt recognition and management of this syndrome in preventing adverse outcomes. Through this report, we aim to contribute to the growing body of literature on PRES, particularly its manifestation and course in pediatric patients with comorbid autoimmune disorders.

## 2 Case Presentation

### 2.1 Case History

A 9‐year‐old child, with a history of celiac disease and dermatitis herpetiformis, presented to the emergency room (ER) exhibiting symptoms of vomiting and loose stools for 2 months, along with a recent seizure lasting 20 min.

### 2.2 Physical Examination

On physical examination, the child appeared pale, emaciated, and drowsy, with a generalized rash. Neurological examination revealed a glasgow coma scale (GCS) score of 10/15, increased muscle tone, and heightened reflexes. The rest of the systemic examination was unremarkable. Upon ER admission, the patient was tachycardic, hypertensive, and had an oxygen saturation of 65%, prompting the initiation of supplemental oxygen and intravenous administration of ceftriaxone, acyclovir, and hydralazine.

### 2.3 Investigations

The patient underwent several laboratory investigations in the ER, which are given below (Tables 1 and 2).

**TABLE 1:** The patient's blood and electrolyte investigations are given in this table.

Investigation	Value	Normal range
Hemoglobin (Hb)	8.8 g/dL	11.5–15.5 g/dL
Hematocrit (Hct)	28.5%	35%–45%
RDW	24.2	12.1%–16.9%
White blood cell (WBC)	17.7 × 10^9^/L	5.0–13.0 × 10^9^/L
Neutrophils	81.6%	40%–75%
Sodium (Na^+^)	135 mmol/L	136–145 mmol/L
Potassium (K^+^)	3.3 mmol/L	3.5–5.1 mmol/L
Chloride (Cl^−^)	98 mmol/L	98–107 mmol/L
Calcium (Ca^+2^)	7.1 mmol/L	8.6–10.2 mmol/L
C‐Reactive protein (CRP)	102.08 mg/L	0–10 mg/L

**TABLE 2:** The patient's blood gases workup is summarized below.

Investigation	Value	Normal range
pH	7.27	7.35–7.45
PCO_2_	55.60 mmHg	27–41
PO_2_	105.10 mmHg	83–108
Bicarbonate	25.10 mEq/L	19–24 mEq/L

**FIGURE 1:**
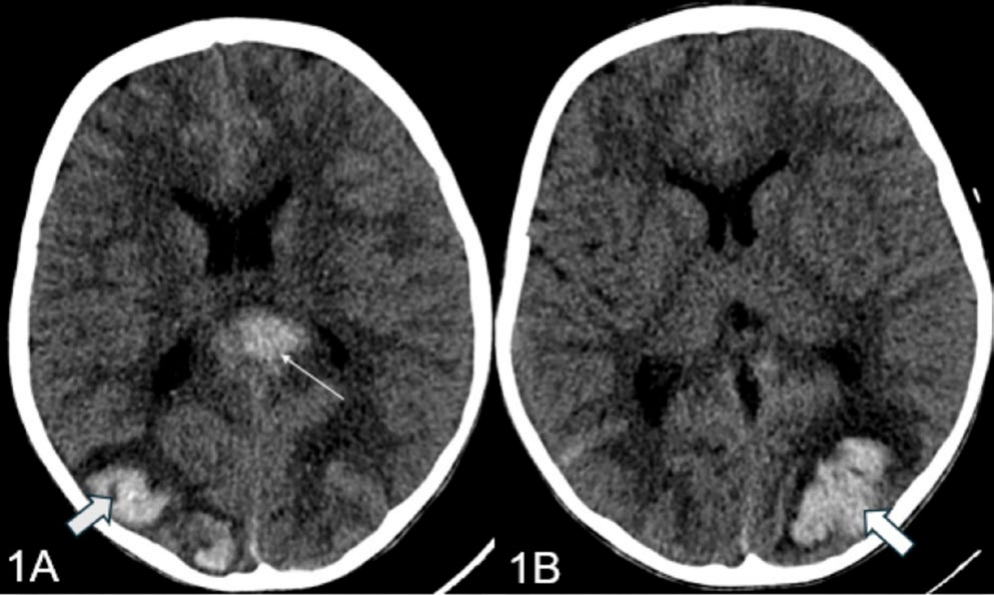
(A, B) Parenchymal hemorrhage with surrounding edema in the occipital lobes and corpus callosum (broad arrows and red arrow).

Subsequently, the patient was transferred to the pediatric intensive care unit (PICU) for further management. In the PICU, the child was intubated following which a CT brain was performed that revealed intraparenchymal and subarachnoid hemorrhages (Figure 1A,B). An extensive workup, including EEG, MRI/MRV/MRA, and prothrombotic tests, was conducted. The patient was administered hypertonic saline, total parenteral nutrition (TPN), Kinz infusion, and methylprednisolone as per hematology's advice. Cardiology consultation revealed a left ventricular ejection fraction (LVEF) of 50%, leading to hydralazine and labetalol infusions. Clotting factor analysis revealed a low Factor XIII level, necessitating transfusions of cryoprecipitate and packed cells. A skin biopsy for immunofluorescence and celiac disease workup was performed, and the patient received treatment for a pseudomonas infection.

### 2.4 Diagnosis

**FIGURE 2:**
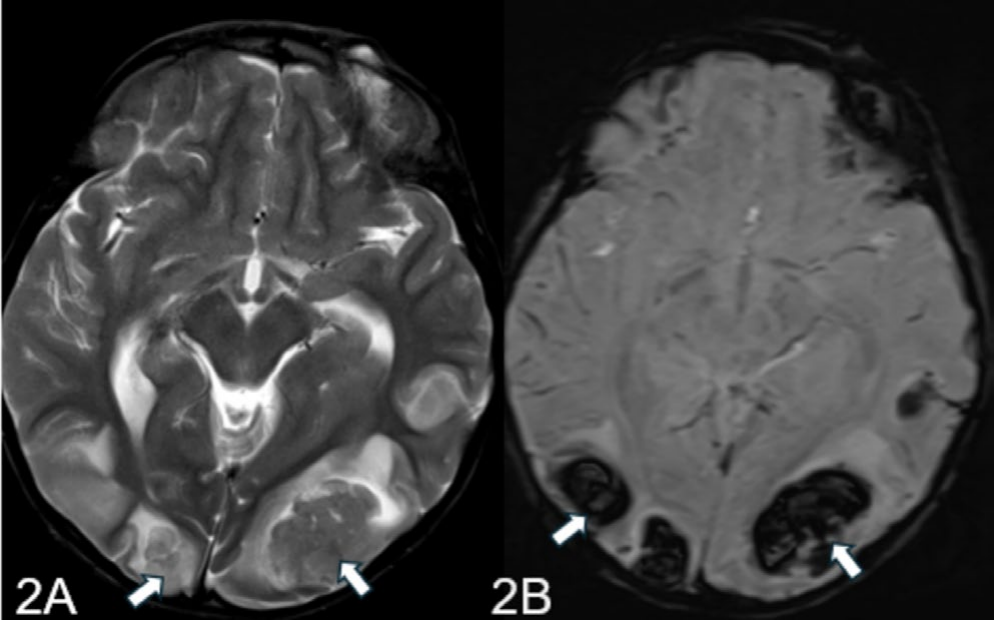
(A, B) MRI shows abnormal T2 signals in the bilateral occipital lobes showing signal drop out on SWI representing hemorrhage.

MRI report of the patient highlighted multifocal areas of parenchymal hemorrhages in the supratentorial region, predominantly at the gray‐white matter junctions in the bilateral parieto‐occipital and parasagittal frontal regions (Figure 2A,B). Subarachnoid hemorrhage components were also noted. The MRA and MRV were unremarkable, with no significant vessel stenosis or filling defects. The findings suggested a diagnosis of PRES, particularly in the context of the patient's history of celiac disease.

### 2.5 Management and Outcome

The patient was extubated 6 days later and transferred to the special care unit (SCU) with a GCS of 7/15. Gradual improvement was noted, with the GCS reaching 13/15. Cardiology reassessment showed an LVEF of 55%, and Aldactone was added to the treatment regimen. Duodenal biopsy results indicated *H. pylori* gastritis, for which a triple regimen was initiated. Rheumatology consultations resulted in the administration of prednisolone and hydroxychloroquine. However, the patient experienced a sudden decline in GCS, accompanied by hypoglycemia, necessitating dextrose boluses and a return to the PICU.

**FIGURE 3:**
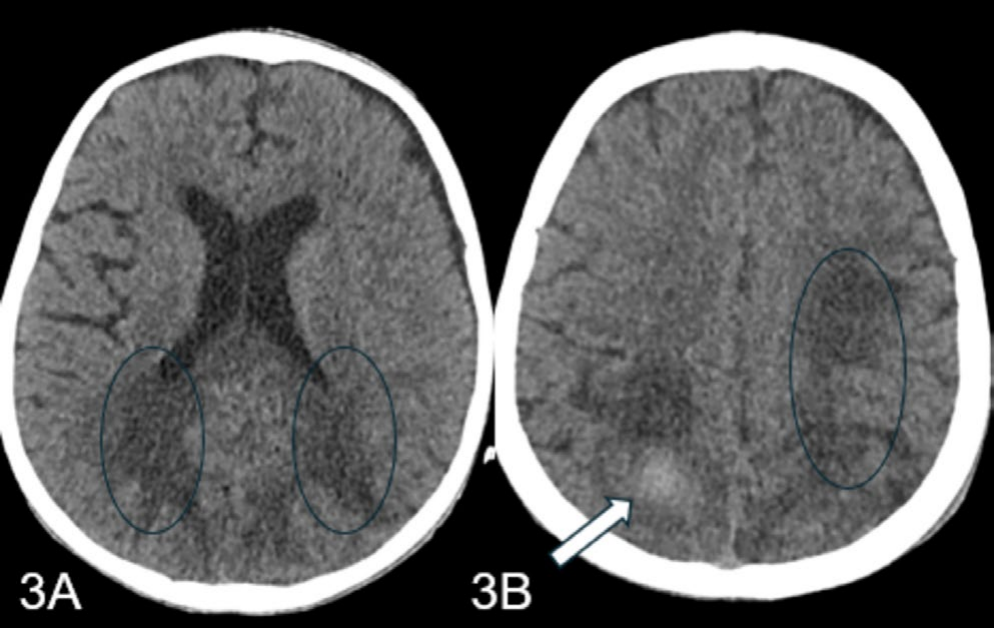
(A, B) Follow up CT scan shows gliosis in the posterior parietal and occipital lobes. Residual hemorrhage in the right posterior parietal lobe (3B arrow).

In the PICU, the child was started on intravenous meropenem, vancomycin, and colistin. A repeat CT head showed resolving bleeds and mild hydrocephalus (Figure 3A,B). Despite a clear coagulation profile and negative ongoing cultures, the child developed acute abdominal pain and distention. An abdominal X‐ray revealed pneumoperitoneum, prompting a surgical consultation. The family was informed of the critical status, and they chose to opt for do not resuscitate (DNR) with comfort care. The child unfortunately went into asystole and was declared dead.

## 3 Discussion

PRES is a neurological disorder characterized by vasogenic edema predominantly in the subcortical white matter of the posterior cerebral hemispheres [2]. Clinically, it manifests with a spectrum of neurological symptoms, including seizures, headaches, visual disturbances, and altered mental status [9]. Despite its name, PRES is not always confined to the posterior regions, nor is it invariably reversible [10].

Etiologically, PRES is associated with several risk factors and triggers. Severe hypertension is a primary risk factor, often precipitating the syndrome by overwhelming the brain's autoregulatory mechanisms [11]. Renal failure, both acute and chronic, can lead to hypertensive crises and electrolyte imbalances, contributing to PRES [12]. Sepsis can cause endothelial dysfunction, which is pivotal in the pathogenesis of PRES [11]. Autoimmune diseases, such as systemic lupus erythematosus, thrombotic thrombocytopenic purpura, and eosinophilic granulomatosis with polyangiitis, are known associations [13]. Additionally, certain immunosuppressive and cytotoxic drugs, including cyclosporine, cisplatin, and cyclophosphamide, have been implicated in the development of PRES [14]. Hypertensive disorders of pregnancy, such as preeclampsia and eclampsia, are also well‐documented triggers [15].

The exact pathophysiology of PRES remains unclear, but two main theories have been proposed. The hypertensive encephalopathy theory suggests that severe hypertension leads to a failure of cerebrovascular autoregulation, resulting in hyperperfusion, endothelial damage, and subsequent vasogenic edema [16]. This theory is supported by observations of elevated or fluctuating blood pressure in the majority of patients with PRES at disease onset [17]. The endothelial dysfunction theory posits that circulating endogenous or exogenous toxins cause direct endothelial injury, leading to the breakdown of the blood–brain barrier, vascular leakage, and edema [18]. This is often observed in patients with conditions like preeclampsia, sepsis, or during treatment with immunosuppressive agents [19].

In celiac disease, gliadin peptides activate CD4+ T cells, triggering release of pro‐inflammatory cytokines (TNF‐α, IFN‐γ, IL‐8) that parallel the cytokine cascade seen in PRES‐associated conditions [20]. This immune activation leads to endothelial dysfunction through multiple convergent pathways: direct cytokine‐mediated endothelial activation (increased ICAM‐1, VCAM‐1, E‐selectin expression), anti‐tTG antibody cross‐reactivity with cerebral endothelium, and systemic inflammatory effects [18]. The compromised intestinal barrier in celiac disease amplifies this process by allowing inflammatory mediators into circulation. The resultant endothelial injury manifests through increased LDH levels, altered vascular tone via competing vasoconstrictive (endothelin‐1, thromboxane‐A2) and vasodilatory (nitric oxide) factors, and blood–brain barrier disruption. VEGF upregulation, triggered by tissue hypoxia and inflammatory cytokines, further increases vascular permeability [14]. The posterior circulation, with reduced sympathetic innervation, is particularly vulnerable to these effects. Celiac‐associated nutritional deficiencies compound endothelial dysfunction: vitamin D deficiency enhances inflammatory responses, magnesium deficiency affects vascular tone (particularly relevant as magnesium acts as a calcium antagonist in cerebral vasculature), and B12 deficiency impairs endothelial function through elevated homocysteine. These deficiencies, combined with chronic inflammation and oxidative stress, create a vulnerability window where additional triggers (infection, blood pressure fluctuations) can precipitate PRES through hypoperfusion/vasoconstriction mechanisms rather than traditional hypertensive theory [21]. This mechanism explains both the rarity of PRES in celiac patients (multiple factors must align) and its potential severity (multiple amplifying pathways). Clinical implications include monitoring for neurological symptoms during active celiac disease and considering celiac screening in cryptogenic PRES cases.

PRES can affect individuals of all ages but is most observed in middle‐aged females [3]. The incidence is likely underreported due to limited awareness and misdiagnosis. Advances in neuroimaging have improved the recognition and diagnosis of PRES, revealing a broader age range and clinical spectrum. Clinically, the symptoms of PRES can vary in severity and typically include seizures, constant generalized headaches, visual disturbances, and altered mental status. Seizures are the most common symptom and can be either generalized tonic–clonic or focal [6]. Headaches are usually constant and generalized, often unresponsive to analgesics. Visual disturbances may include visual hallucinations, cortical blindness, hemianopia, and diplopia [2]. Altered mental status can range from confusion and agitation to stupor and coma. Other symptoms may include nausea, vomiting, and occasionally focal neurological deficits [22].

The diagnosis of PRES relies heavily on neuroimaging and clinical assessment. MRI of the brain is the gold standard, typically showing bilateral vasogenic edema in the posterior cerebral hemispheres, particularly in the parieto‐occipital regions, although other regions can be involved [3]. CT scans are often used initially to rule out acute emergencies like hemorrhage. Lumbar puncture may be performed to exclude other causes of altered mental status, with cerebrospinal fluid findings usually normal or showing mildly elevated protein [23]. EEG may be used to detect nonconvulsive seizures or status epilepticus [6].

Management of PRES involves addressing the underlying cause while providing symptomatic treatment [21]. Blood pressure control is critical, with a recommended initial reduction of 25% from baseline, avoiding rapid fluctuations to prevent complications [24]. Anticonvulsants are administered for seizure control, with tapering off when symptoms resolve. If PRES is associated with immunosuppressive or cytotoxic drugs, careful dose reduction or discontinuation should be considered, depending on the patient's condition [25]. Supportive care includes maintaining high normal magnesium levels, managing potential cerebral vasospasm, and addressing electrolyte imbalances, infections, and nutritional needs [26]. Initial treatment focuses on aggressive strategies to reduce mass effect and edema using steroids, hyperosmolar therapy, and moderate blood pressure control. In cases involving autoimmune conditions, immunomodulator treatments, including steroids and cyclophosphamide, have shown effectiveness. While specific protocols for PRES in the context of celiac disease are not detailed, the general principle of treating both the acute PRES manifestations and the underlying autoimmune condition remains paramount.

The prognosis for PRES is generally favorable, with most patients experiencing complete recovery within days to weeks if promptly and appropriately treated. However, delayed treatment can result in permanent neurological deficits or death [27]. Long‐term complications may include chronic epilepsy or recurrent PRES episodes [28]. Conditions to consider in the differential diagnosis include intracranial hemorrhage, subarachnoid hemorrhage, cerebral venous sinus thrombosis, posterior circulation ischemic stroke, central nervous system vasculitis, encephalitis, and severe hypoglycemia. Although the specific relationship between celiac disease and PRES has not been extensively studied in the literature, general principles about autoimmune conditions and PRES outcomes may provide insight. As an autoimmune disorder, celiac disease likely influences PRES through similar inflammatory mechanisms documented in other autoimmune conditions, where endothelial dysfunction occurs through cytokine activation (TNFα, IL‐1, and IFNγ). The overall prognosis of PRES is generally favorable, with complete recovery in 75%–90% of patients and mortality rates of 3%–6%. However, autoimmune conditions can potentially complicate the course through increased inflammatory responses and vascular permeability. In celiac disease specifically, the underlying chronic inflammation and potential nutritional deficiencies could theoretically impact the brain's ability to recover from the vasogenic edema characteristic of PRES. Management would likely require not only standard PRES treatments but also strict attention to gluten‐free diet adherence and nutritional status to optimize outcomes. As with other autoimmune conditions, the degree of disease control may influence recovery. However, specific research is needed to confirm these theoretical relationships, as the exact impact of celiac disease on PRES outcomes remains to be determined through dedicated clinical studies.

The presented case of a 9‐year‐old child with a history of celiac disease and dermatitis herpetiformis exemplifies the complexity and severity of PRES. The child's clinical presentation with seizures, altered mental status, and vomiting over 2 months, coupled with significant hypertension, aligns with the typical features of PRES. The patient's underlying celiac disease likely contributed to a pro‐inflammatory state, exacerbating the risk for PRES. Severe hypertension observed upon ER admission was also a critical risk factor, overwhelming the brain's autoregulatory capacity and leading to vasogenic edema. The presence of multifocal parenchymal hemorrhages further underscores the severity of endothelial dysfunction in this case. Despite aggressive management, including antihypertensives, seizure prophylaxis, and supportive care, the child's condition deteriorated, illustrating the potential severity of PRES and the importance of early recognition and comprehensive management.

## 4 Conclusion

This case report highlights the critical importance of considering PRES in pediatric patients with autoimmune conditions, particularly celiac disease, who present with acute neurological symptoms. The atypical presentation and rapid progression to a fatal outcome underscore the need for heightened vigilance and prompt intervention in such cases. Although the association between celiac disease and PRES is not well established, this case suggests a potential link that warrants further investigation. Moving forward, clinicians should maintain a high index of suspicion for PRES in pediatric patients with celiac disease, even when typical radiological findings are absent. This approach may facilitate earlier diagnosis and treatment, potentially improving outcomes in this vulnerable patient population. Future research should focus on elucidating the mechanisms underlying the possible connection between celiac disease and PRES, as well as developing targeted management strategies for this challenging clinical scenario.

## Author Contributions

**Hashim Salar:** conceptualization, data curation, visualization, writing – original draft, writing – review and editing. **Khizer Masroor Anns:** supervision, validation, visualization, writing – review and editing. **Musa Salar:** validation, visualization, writing – original draft, writing – review and editing. **Muhammad Aman:** validation, visualization, writing – review and editing. **Faheemullah Khan:** project administration, resources, supervision, validation, visualization, writing – review and editing. **Uffan Zafar:** supervision, visualization. **Izaz Ahmad:** validation, visualization. **Sundas Basharat:** supervision, validation, visualization. **Rehana Murad:** validation, visualization, writing – review and editing. **Khizar Salar:** supervision, writing – review and editing. **Shayan Sirat Maheen Anwar:** validation, visualization, writing – review and editing.

## Ethics Statement

Ethical approval was not required because it was a case report.

## Consent

Written informed consent was taken from the patient for this case report prior to publishing it. Final manuscript was reviewed by all the authors, and consent was taken prior to publishing it.

## Conflicts of Interest

The authors declare no conflicts of interest.

## Data Availability

Data pertaining to this case report can be procured from the corresponding author upon request.
